# Intra and Inter-rater Reliability between Ultrasound Imaging and Caliper Measures to determine Spring Ligament Dimensions in Cadavers

**DOI:** 10.1038/s41598-019-51384-6

**Published:** 2019-10-15

**Authors:** Fernando Santiago-Nuño, Patricia Palomo-López, Ricardo Becerro-de-Bengoa-Vallejo, César Calvo-Lobo, Marta Elena Losa-Iglesias, Israel Casado-Hernández, Daniel López-López

**Affiliations:** 1 Research, Health and Podiatry Unit. Department of Health Sciences, Faculty of Nursing and Podiatry. Universidade da Coruña, Ferrol, Spain; 2 University Center of Plasencia. Universidad de Extremadura, Badajoz, Spain; 3 Facultad de Enfermería, Fisioterapia y Podología, Universidad Complutense de Madrid, Madrid, Spain; 4 Faculty of Health Sciences, Universidad Rey Juan Carlos, Móstoles, Spain

**Keywords:** Ligaments, Muscle

## Abstract

The purpose was to evaluate intra and inter-rater reliability, repeatability and absolute accuracy between ultrasound imaging (US) and caliper measures to determine Spring ligament (SL) dimensions in cadavers. SLs were identified from 62 human feet from formaldehyde-embalmed cadavers. Intra and inter-observer reliability, repeatability and absolute accuracy of SL width, thickness and length between US and caliper measurements were determined at intra and inter-session by intraclass correlation coefficients, Pearson´s correlation coefficients, Student *t* tests, standard errors of measurement, minimum detectable changes, values of normality, 95% limits of agreement, and Bland-Altman plots. Excellent inter-session and inter-rater reliability, adequate absolute accuracy, almost perfect agreement and strong correlations were shown for caliper, US and their comparison for all SL dimensions. US measurements presented higher absolute accuracy than caliper measures for SL length and thickness dimensions, while caliper displayed greater absolute accuracy for SL width dimensions. Good repeatability (*P* > 0.05) was shown for all SL dimensions by US, caliper and their comparison, except for SL width dimension measured with US (*P* = 0.019). Both US and caliper could be recommended for all SL dimensions evaluation due to their excellent reliability and absolute accuracy in cadavers, although width dimensions should be considered with caution due to US repeatability differences.

## Introduction

Spring ligament (SL), also called as plantar calcaneonavicular ligament, plays an important role as the main stabilizer of the foot internal arch with great repercussion in the hindfoot function^1^. In addition, secondary structures such as plantar fascia, superficial deltoid ligament and other plantar ligaments in conjunction with the SL may be considered as the responsible anatomic structures to maintain the foot internal arch. SL is extended from the calcaneus to navicular bones forming a sling that stabilizes the hindfoot^2^.

Indeed, SL injuries seem to be very common and related to the rupture of the ligament, leading to plantar flexion talus displacement and valgus hindfoot deformity, which may generate an adult acquired pes planus. Consequently, surgery may be required for this rupture after traumatic conditions or others factors such as degenerative disease, iatrogenic injury, infection or tumors on the hindfoot, which require a full understanding of the SL anatomy. In addition, SL reconstruction provides a good correction of the foot internal arch with main implications in the rehabilitation field. Accordingly, anatomic landmarks of the SL may be essential to play down surgery-associated lesions and deformities of the hindfoot^3,4^. Several studies have evaluated this anatomic structure by ultrasound imaging (US) and caliper measurements in cadavers^5–8^, although prior investigations have not yet analyzed the reliability for measuring SL dimensions using caliper and US to determine the anatomic correlation of the SL in cadaveric feet. Thus, given that there is existing literature correlating ultrasound measurements to clinical decisions and other imaging modalities^5–8^, absolute accuracy of the measurement needs to be determined due to the lack of studies about reliability and correlation between caliper and US measurements of the SL in cadaveric feet addressing width, thickness and length for a better accuracy of these evaluations and the improvement of ultrasound-guided procedures^9^. Both intra and inter-rater reliability needs to be detailed in order to determine absolute accuracy and repeatability of these measurements within a same evaluator and between both evaluators by both US and caliper^10–13^. Separately, both tools have shown appropriate reliability for SL dimensions measured by experienced raters, nevertheless US and caliper measures have not been compared as well as SL width dimensions have not yet been measured by US^5–8^. We hypothesized that caliper and US use may show an excellent reliability to analyze of the SL anatomic dimensions in cadaver. Thus, the study purpose was to evaluate the intra and inter-rater reliability between US and caliper measures to determine SL width, thickness and length in cadaveric feet at intra and inter-session.

## Materials and Methods

### Study design

A reliability study was carried out in order to determine the intra and inter-rater reliability between US and caliper measures detailing SL width, thickness and length in cadaveric feet at intra and inter-session. The Updated List of Essential Items for Reporting Diagnostic Accuracy Studies (STARD 2015) criteria were followed^14^.

### Sample size calculation

The minimum number of specimens required was calculated based on reliability testing to determine reliability. In this study, the ICCs were used for reliability testing at a target value of 0.8 and a 95% CI of 0.2. We calculated the sample size to be 36 specimens with a Bonett’s approximation^15^.

### Ethical aspects

This research was approved by the local Research Ethical Committee in the University of Rey Juan Carlos (URJC), in the town of Móstoles, province of Madrid (Spain), with internal code 0801201800618. In addition, all methods were performed in accordance with the relevant guidelines and regulations. Consent for this study was previously obtained from the anatomy department.

### Cadavers and embalming method

Sixty-two feet from formaldehyde-embalmed human cadavers, 8 males and 26 females, without presence of any type of trauma were recruited in our research protocol^9^. The mean (SD) age was 76.46 (6.46) years; range from 66 to 89 years old. The human cadaveric feet comprised 30 right and 32 left feet. The adult cadavers came from the Scientific Anatomy Center, S.L. in the town of Valencia (Spain) in the town of Valencia (Spain). Scientific Anatomy Center, S.L. which included informed consent as part of the cadaver donation process.

The used preservation method for embalming the cadavers was perfusion through the femoral artery with a blending of formaldehyde, ethanol, methanol, phenol and glycerine that improve the longevity of the body and tissues, reducing the infection risk^16^.

### Ultrasound measurements

US images were recorded by a Mindray Z6 Digital Ultrasonic Diagnostic System (Shenzhen Mindray Bio-Medical Electronics Co., Ltd, Shenzhen, China) by using a linear transducer type L4-P with a frequency bandwidth range of 5–10 MHz.

**Figure 1:**
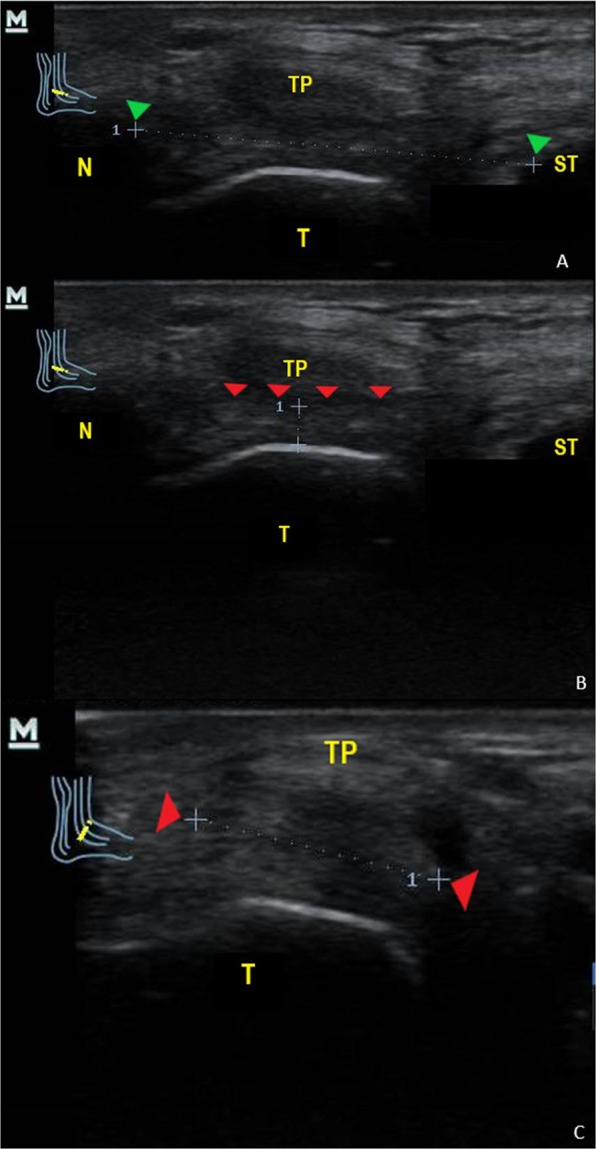
Ultrasound measurements of the Spring Ligament for length (**A**), thickness (**B**) and width (**C**) dimensions in cadaver foot. *Abbreviations:* N, Navicular; ST; Sustentaculum Tali; T, Talus; TP, Tibial Posterior tendon. Green arrows showed bone references of Navicular and Sustentaculum Tali for length measurements. Red arrows showed Spring Ligament references for thickness and width measurements.

All human cadaver feet were located at the same immobilized neutral position. Then, two independent and experienced musculoskeletal podiatrists (with at least 5 years of musculoskeletal US experience) collected the US measurements to determine the width, thicknesses and length (cm) of the SL in cadaveric feet (Fig. 1).

### Caliper measurements

**Figure 2:**
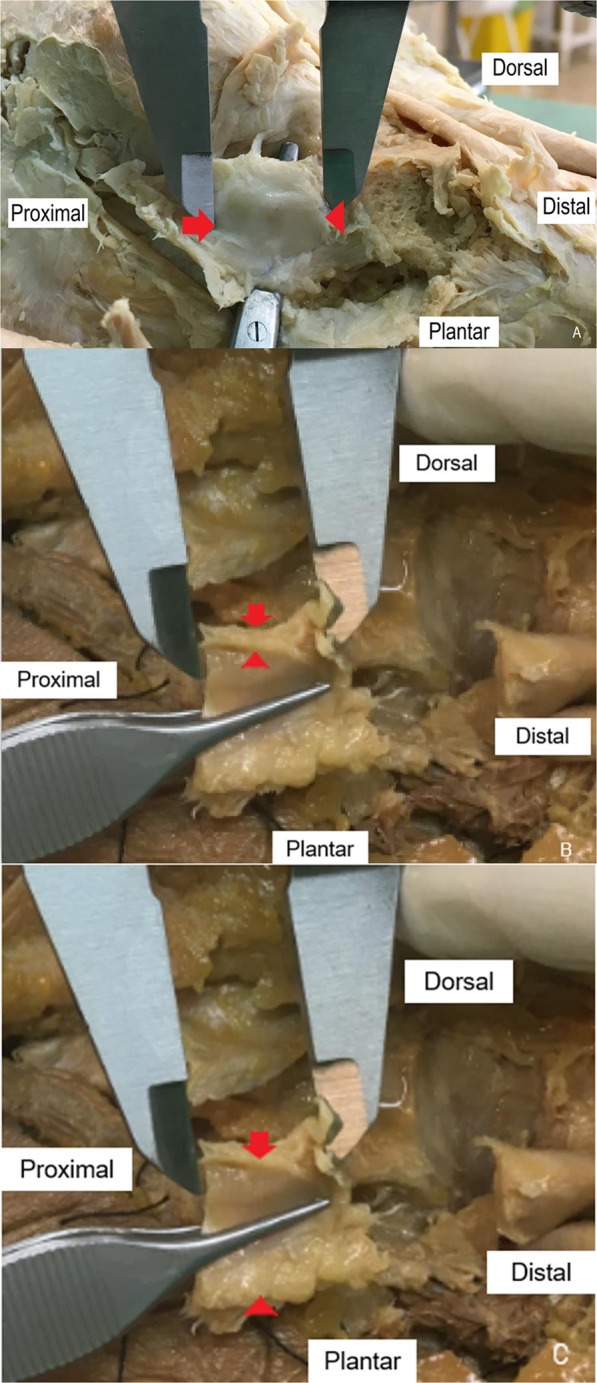
Caliper measurements of the Spring Ligament for length (**A**), thickness (**B**) and width (**C**) dimensions in cadaver foot. Red arrows showed Spring Ligament references for thickness and width measurements.

Thereafter, the foot cadaver dissection was carried out in order to expose the SL for its measurement with a digital LCD caliper (BURG-WÄCHTER KG, Wetter, Germany) with the subtalar joint foot in neutral position. Two podiatrists recorded the width, thicknesses and length (cm) of the SL in cadaveric feet with this device (Fig. 2).

### Reliability study protocol

After two days, the protocol design was repeated identical to the first session of measure. The values of the measurements from 1^st^ and 2^nd^ sessions as well as 1^st^ and 2^nd^ observers were used to analyze the intra and inter-rater reliability at intra and inter-session. The podiatrists did not have access to the information records of the 1^st^ session until recorded values were registered after the 2^nd^ session.

### Statistical analyses

Statistical analyses were carried out by the statistical package of SPSS 19.0 software for windows (SPSS Inc., Chicago, USA). First, the Kolmogorov-Smirnov test was used to assess normality. All variables were parametric data due to a normal distribution was shown (according to a *P*-value > 0.05 of the Kolmogorov-Smirnov test). Second, mean ± standard deviation (SD) as well as upper and lower limits for 95% confidence interval (CI) were used in order to describe all data. Finally, differences between two measurement values were analyzed by the Student *t* test for paired samples.

Reliability between two measurement values was determined by the Intraclass Correlation Coefficient (ICC) and Pearson´s correlation coefficient (*r*). Indeed, ICC values were interpreted as poor (ICC < 0.40), fair (ICC = 0.40–0.59), good (ICC = 0.60–0.74), and excellent (ICC = 0.75–1.0)^17^. In addition, *r* coefficient values were categorized as weak (*r* = 0.00–0.40), moderate (*r* = 0.41–0.69), and strong (*r* = 0.70–1.00)^18^.

The 95% limits of agreement (LoA) between sessions and devices expressed the degree of error proportional to the mean of the measurement units, and these statistics were calculated using the methods described by Bland and Altman^11^. If the differences between the measurements tended to agree, the results were close to zero.

Standard errors of measurement (SEM) were calculated to measure the range of error of each parameter. The SEM was calculated from the ICCs and SDs for each of the three measurements. SEM were calculated according to the formula SEM = SD × sqrt (1 − ICC). Indeed, the minimum detectable change (MDC) was calculated from the SEM values by the following formula MDC = $\sqrt{2}\times 1.96\times SEM$ at a 95% CI which reflected the magnitude of change necessary to provide confidence to be sure about these changes were not the result of random variations or measurement errors. Both SEM and MDC were analyzed according to Bland and Altman^12^. Furthermore, values of normality (VN) of the sample for all outcome measurements were obtained by the formula VN = Mean + /_1.96 * SD.

Finally, Bland-Altman plots^11,12^ were calculated to display the agreement between US and caliper. These plots showed the difference between each pair of measurements on the y-axis against the mean of each pair of measurements on the x-axis. A *P*-value < 0.05 with a 95% CI was used for the data analysis.

## Results

Analysis of reliability of the SL morphology by US between the first and second session by first observer (Table 1) showed excellent intra-rater (ICC_(1-1)_ = 0.992–1.00) and inter-rater reliability (ICC_(1-1)_ = 0.997–0.999) with a strong correlation (*r* = 0.994–0.998; *P* < 0.05) for length and thickness measurements. Nevertheless, poor to good intra-rater (ICC_(1-1)_ = 0.545–0.612) and inter-rater reliability (ICC_(1-1)_ = 0.279) with a weak non-significant correlation (*r* = −0.124; *P* > 0.001) was shown for width measurements. In addition, there were not statistically significant differences (*P* < 0.05) between sessions.

**Table 1:** Analysis of reliability of the Spring Ligament dimensions by ultrasound between the first and second session by first observer and normalized values.

Sessions	First Observer Us Measurements																	
	First Session					Second Session					Intersession							
Variables	Mean (SD) 95% CI	ICC_(1-1)_ (95% CI)	SEM	MDC	Normalized values (95% CI)	Mean (SD) 95% CI	ICC_(1-1)_ (95% CI)	SEM	MDC	Normalized values (95% CI)	Mean (SD) 95% CI	ICC (95% CI)	SEM	MDC	*LoA (95%CI)*	*P*-value	*r* (*P*-value)	Normalized values (95% CI)
Length	1.57 (0.33) 1.48–1.65	0.995 (0.993–0.997)	0,0009	0,002	1.57 (0.64) (0.93–2.21)	1.57 (0.33) 1.49–1.65	1 (0.999–1)	0.000	0.0001	1.57 (0.64) (0.93–2.21)	1.57 (0.33) 1.49–1.65	0.999 (0.998–0.999)	0.0001	0,0003	0–00 (−0.04–0.04)	0.118	0.998 (<0.001)	1.57 (0.64) (0.93–2.21)
Thickness	0.44 (0.10) 0.41–0.46	0.992 (0.988–0.995)	0.008	0.024	0.46 (0.19) (0.27–0.65)	0.44 (0.10) 0.41–0.46	0.994 (0.991–0.996)	0,007	0.021	0.44 (0.19) (0.25–0.63)	0.44 (0.10) 0.41–0.46	0.997 (0.995–0.998)	0.005	0.015	0.00 (−0.02–0.02)	0.740	0.994 (<0.001)	0.44 (0.19) (0.25–0.63)
Width	1.25 (0.14) 1.21–1.28	0.612 (0410–0753)	0.087	0.241	1.25 (0.27) (0.98–1.52)	1.26 (0.11) 1.23–1.29	0.545 (0.308–0.710)	0.074	0.205	1.25 (0.14) (1.21–1.28)	1.25 (0.00) 1.24–1.26	0.279 (−0.305–0.519)	0	0	−0.01 (−0.40–0.38)	0.634	−0.124 (0.336)	1,25 (0,15) (1.10–1.40)

*Abbreviations*: CI, confidence interval; ICC, Intraclass Correlation Coefficient; LoA, 95% limits of agreement; MDC, Minimum Detectable Change; *r*, Pearson correlation coefficient; SEM, standard error of measurement; SD, standard deviation; US, ultrasound imaging.

Analysis of reliability of the SL dimensions by caliper between the first and second session by first observer (Table 2) showed excellent intra-rater (ICC_(1-1)_ = 0.875–1.00) and inter-rater reliability (ICC_(1-1)_ = 0.958–0.996) with a strong correlation (*r* = 0.922–0.992; *P* < 0.001) for length, thickness and width measurements. In addition, there were not statistically significant differences (*P* > 0.05) between sessions.

**Table 2:** Analysis of reliability of the Spring Ligament dimensions by caliper between the first and second session by first observer and normalized values.

Sessions	First Observer Caliper Measurements																	
	First Session					Second Session					Intersession							
Variables	Mean (SD) 95% CI	ICC_(1-1)_ (95% CI)	SEM	MDC	Normalized values (95% CI)	Mean (SD) 95% CI	ICC_(1-1)_ (95% CI)	SEM	MDC	Normalized values (95% CI)	Mean (SD) 95% CI	ICC (95%CI)	SEM	MDC	*LoA (95%CI)*	*P*-value	*r* (*P*-value)	Normalized values (95% CI)
Length	1.68 (0.62) 1.52–1.83	1 (1–1)	0.000	0.000	1.58 (0.68) (0.9–2.26)	1.59 (0.30) 1.50–1.67	1 (1–1)	0	0	1.59 (0.58) (1.01–2.17)	1.63 (0.06) (1.54–1.72)	0.959 (0.931–0.975)	0.0436	0.1209	0.10 (−1.00–1.20)	0.690	0.929 (<0.001)	1.59 (0.62) (0.97–2.21)
Thickness	0.41 (0.10) 0.38–0.44	0.999 (0.999–0.999)	0.003	0.008	0.41 (0.19) (0.22–0.60)	0.41 (0.10) 0.38–0.44	0.989 (0.983–0.993)	0.010	0.029	0.41 (0.19) (0.22–0.60)	0.41 (0.10) 0.38–0.44	0.996 (0.994–0.998)	0.006	0.017	0.00 (−0.03–0.02)	0.694	0.992 (<0.001)	0.41 (0.19) (0.22–0.60)
Width	1.21 (0.10) 1.19–1.24	0.875 (0.810–0.921)	0.035	0.098	1,23 (0,18) (1.19–1.28)	1.22 (0.11) 1.19–1.24	0.999 (0.999–0.999)	0.003	0.009	1.21 (0.21) (1–1.42)	1.21 (0.00) 1.19–1.24	0.958 (0.930–0.975)	0	0	0.00 (−0.04–0.03)	0.133	0.922 (<0.001)	1.21 (1–1.42)

*Abbreviations*: CI, confidence interval; ICC, Intraclass Correlation Coefficient; LoA, 95% limits of agreement; MDC, Minimum Detectable Change; *r*, Pearson correlation coefficient; SEM, standard error of measurement; SD, standard deviation.

Analysis of reliability of the SL dimensions by US between the first and second session by second observer (Table 3) showed excellent intra-rater (ICC_(1-1)_ = 0.987–0.999) and inter-rater reliability (ICC_(1-1)_ = 0.995–0.998) with a strong correlation (*r* = 0.991–0.996; *P* < 0.001) for length and thickness measurements. Nevertheless, poor to fair intra-rater (ICC_(1-1)_ = 0.276–0.540) and inter-rater reliability (ICC_(1-1)_ = 0.213) with a weak non-significant correlation (*r* = 0.124; *P* > 0.05) was shown for width measurements. In addition, there were not statistically significant differences (*P* < 0.05) between sessions.

**Table 3:** Analysis of reliability of the Spring Ligament dimensions by ultrasound between the first and second session by second observer and normalized values.

Sessions	Second Observer Us Measurements																	
	First Session					Second Session					Intersession							
Variables	Mean (SD) 95% CI	ICC_(1-1)_ (95% CI)	SEM	MDC	Normalized values (95% CI)	Mean (SD) 95% CI	ICC_(1-1)_ (95% CI)	SEM	MDC	Normalized values (95% CI)	Mean (SD) 95% CI	ICC (95%CI)	SEM	MDC	*LoA (95%CI)*	*P*-value	*r* (*P*-value)	Normalized values (95% CI)
Length	1.57 (0.32) 1.49–1.66	0.999 (0.999–1)	0.0001	0.0003	1.57 (0.60) (0.95–2.17)	1.57 (0.32) 1.49–1.65	0.994 (0.990–0.996)	0.0009	0.0025	1.57 (0.62) (0.95–2.19)	1.57 (0.32) (1.49–1.65)	0.998 (0.997–0.999)	0	0.0001	0.00 (−0.03–0.03)	0.734	0.996 (<0.001)	1.57 (0.62) (0.95–2.19)
Thickness	0.44 (0.09) 0.41–0.46	0.997 (0.995–0.998)	0.004	0.013	0.44 (0.17) (0.27–0.61)	0.44 (0.09) 0.41–0.46	0.987 (0.980–0.992)	0.010	0.028	0.44 (0.17) (0.27–0.61)	0.44 (0.09) 0.41–0.46	0.995 (0.992–0.997)	0.007	0.019	0.00 (−0.06–0.06)	0.772	0.991 (<0.001)	0.44 (0.17) (0.27–0.61)
Width	1.24 (0.11) 1.21–1.27	0.276 (−0.109–0.542)	0.093	0.259	1.22 (0.25) (0.97–1.45)	1.20 (0.10) 1.17–1.22	0.540 (0.293–0.709)	0.067	0.188	1.18 (0.23) (0.95–1.48)	1.22 (0.07) 1.20–1.24	0.213 (−0,276–0.519)	0.062	0.172	0.04 (−0.25–0.33)	0.063	0.124 (0.338)	1.20 (0.19) (1.01–1.39)

*Abbreviations*: CI, confidence interval; ICC, Intraclass Correlation Coefficient; LoA, 95% limits of agreement; MDC, Minimum Detectable Change; *r*, Pearson correlation coefficient; SEM, standard error of measurement; SD, standard deviation; US, ultrasound imaging.

Analysis of reliability of the SL dimensions by caliper between the first and second session by second observer (Table 4) showed excellent intra-rater (ICC_(1-1)_ = 0.877–1.00) and inter-rater reliability (ICC_(1-1)_ = 0.996) with a strong correlation (*r* = 0.936–0.993; *P* < 0.001) for length, thickness and width measurements. In addition, there were statistically significant differences (*P* < 0.05) between sessions for length, but not for thickness or width measurements (*P* > 0.05).

**Table 4:** Analysis of reliability of the Spring Ligament dimensions by caliper between the first and second session by second observer and normalized values.

Sessions	Second Observer Caliper Measurements																	
	First Session					Second Session					Intersession							
Variables	Mean (SD) 95% CI	ICC_(1-1)_ (95% CI)	SEM	MDC	Normalized values (95% CI)	Mean (SD) 95% CI	ICC_(1–1)_ (95% CI)	SEM	MDC	Normalized values (95% CI)	Mean (SD) 95% CI	ICC (95% CI)	SEM	MDC	*LoA* *(95%CI)*	*P*-value	*r* (*P*-value)	Normalized values (95% CI)
Length	1.58 (0.32) 1.50–1.66	1 (1–1)	0	0	1.58 (0.62) (0.96–2.20)	1.59 (0.31) 1.51–1.67	1 (1–1)	0	0	1.59 (0.60) (0.99–2.19)	1.58 (0.32) (1.50–1.66)	0.996 (0.994–0.998)	0,0004	0,0012	−0.01 (−0.08–0.06)	0.039	0.993 (<0.001)	1.58 (0.62) (0.96–2.20)
Thickness	0.41 (0.10) 0.38–0.43	0.987 (0.980–0.992)	0.011	0.31	0.41 (0.19) (0.22–0.60)	0.41 (0.10) 0.39–0.44	0.999 (0.999–1)	0.003	0.008	0.41 (0.19) (0.22–0.60)	0.41 (0.10) 0.38–0.44	0.996 (0.993–0.997)	0.006	0.017	−0.00 (−0.03–0.02)	0.454	0.992 (<0.001)	0.41 (0,19) (0.22–0.60)
Width	1.22 (0.11) 1.19–1.24	0,999 (0.999–0.999)	0.003	0.009	1,22 (0,21) (1.01–1.43)	1.22 (0.13) 1.19–1.25	0.999 (0.999–0.999)	0.004	0.011	1.20 (0.25) (0.95–1.45)	1.22 (0.11) 1.19–1.24	0.999 (0.999–0.999)	0.003	0.009	−0.001 (−0.006–0.004)	0.392	0.936 (<0.001)	1.21 (0,21) (1–1.42)

*Abbreviations*: CI, confidence interval; ICC, Intraclass Correlation Coefficient; LoA, 95% limits of agreement; MDC, Minimum Detectable Change; *r*, Pearson correlation coefficient; SEM, standard error of measurement; SD, standard deviation.

Analysis of reliability of the SL dimensions by first observer between US and caliper measurements (Table 5) showed excellent intra-rater reliability (ICC_(1-1)_ = 0.877–0.978) with a strong correlation (*r* = 0.805–0.957; *P* < 0.001) for length and thickness measurements. Nevertheless, poor intra-rater reliability (ICC_(1-1)_ = 0.207) with a weak non-significant correlation (*r* = 0.127; *P* > 0.05) was shown for width measurements. In addition, there were inter-session statistically significant differences (*P* < 0.05) between US and caliper measurements for thickness and width, but not for length measurements (*P* > 0.05).

**Table 5:** Analysis of reliability of the Spring Ligament dimensions by first observer between ultrasound and caliper measurements and normalized values.

Obserber	First Observer Us measurements	First Observer Caliper measurements	Intersession							
Variables	Mean (SD) 95% CI	Mean (SD) 95% CI	Mean (SD) 95% CI	ICC_(1-1)_ (95% CI)	SEM	MDC	*LoA* *(95%CI)*	*P*-value	*r* (*P*-value)	Normalized values (95% CI)
Length	1.57 (0.33) 1.49–1.65	1.63 (0.06) (1.54–1.72)	1.58 (0,33) 1.49–1.66	0.978 (0.963–0.986)	0.0196	0.0542	0.06 (−0.50–0.63)	0.170	0.957 (<0.001)	1.58 (0.64) (0.94–2.22)
Thickness	0.44 (0.10) 0.41–0.46	0.41 (0.10) 0.38–0.44	0.42 (0.10) 0.40–0.45	0.877 (0.773–0.931)	0.035	0.097	0.06 (−0.50–0.63	0.002	0.805 (<0.001)	0.42 (0.19) (0.23–0.61)
Width	1.25 (0.00) 1.24–1.26	1.21 (0.00) 1.19–1.24	1.23 (0.02) 1.19–1.26	0.207 (−0.265–0.510)	0.017	0.049	0.03 (−0.10–0.15)	0.018	0.127 (0.327)	1.23 (0.19) (1.04–1.42)

*Abbreviations*: CI, confidence interval; ICC, Intraclass Correlation Coefficient; LoA, 95% limits of agreement; MDC, Minimum Detectable Change; *r*, Pearson correlation coefficient; SD, standard deviation; US, ultrasound imaging.

Analysis of reliability of the SL dimensions by second observer between US and caliper measurements (Table 6) showed excellent intra-rater reliability (ICC_(1-1)_ = 0.862–0.996) with a strong correlation (*r* = 0.781–0.993; *P* < 0.001) for length and thickness measurements. Nevertheless, poor intra-rater reliability (ICC_(1-1)_ = 0.232) with a weak non-significant correlation (*r* = −0.104; *P* > 0.05) was shown for width measurements. In addition, there were inter-session statistically significant differences (*P* < 0.05) between US and caliper measurements for thickness, but not for length and width measurements (*P* > 0.05).

**Table 6:** Analysis of reliability of the Spring Ligament dimensions by second observer between ultrasound and caliper measurements and normalized values.

Obserber	Second Observer Us Measurements	Second Observer Caliper Measurements	Intersession							
Variables	Mean (SD) 95% CI	Mean (SD) 95% CI	Mean (SD) 95% CI	ICC_(1-1)_ (95% CI)	SEM	MDC	*LoA (95%CI)*	*P*-value	*r* (*P*-value)	Normalized values (95% CI)
Length	1.57 (0.33) 1.49–1.65	1.58 (0.32) 1.50–1.66	1.58 (0.32) 1.50–1.66	0.996 (0.993–0.998)	0.0004	0.0011	0.01 (−0.07–0.09)	0.084	0.993 (<0.001)	1.58 (0,64) (0.94–2.22)
Thickness	0.44 (0.09) 0.41–0.46	0.41 (0.10) 0.38–0.44	0.42 (0.10) 0.40–0.45	0.862 (0.752–0.921)	0.037	0.103	0.03 (−0.16–0.10)	0.003	0.781 (<0.001)	0.42 (0.19) (0.23–0.61)
Width	1.22 (0.07) 1.20–1.24	1.22 (0.11) 1.19–1.24	1.22 (0.00) 1.22–1.22	´0.232 (−1.068–0.263)	0	0	0.00 (−0.27–0.27)	0.709	´−0.104 (0.422)	1.21 (0.21) (1–1.42)

*Abbreviations*: CI, confidence interval; ICC, Intraclass Correlation Coefficient; LoA, 95% limits of agreement; MDC, Minimum Detectable Change; *r*, Pearson correlation coefficient; SD, standard deviation; US, ultrasound imaging.

Analysis of reliability of the SL dimensions by US between inter-session first and second observer (Table 7) showed excellent inter-rater reliability (ICC_(1-1)_ = 0.938–0.994) with a strong correlation (*r* = 0.893–0.989; *P* < 0.001) for length, thickness and width measurements. Nevertheless, there were inter-rater statistically significant differences (*P* < 0.05) between first and second observer for width measurements, but not for length and thickness measurements (*P* > 0.05).

**Table 7:** Analysis of reliability of the Spring Ligament dimensions by ultrasound between inter-session first and second observer and normalized values.

Obserber	First Observer Intersession	Second Observer Intersession	Intersession							
Variables	Mean (SD) 95% CI	Mean (SD) 95% CI	Mean (SD) 95% CI	ICC_(1-1)_ (95% CI)	SEM	MDC	*LoA (95%CI)*	*P*-value	*r* (*P*-value)	Normalized values (95% CI)
Length	1.57 (0.33) 1.49–1.65	1.57 (0.32) 1.49–1.65	1.57 (0.32) 1.49–1.65	0.994 (0.991–0.996)	0.025	0.069	−0.01 (−0.12–0.10)	0.922	0.989 (<0.001)	1.57 (0.62) (0.95–2.19)
Thicknesses	0.44 (0.10) 0.41–0.46	0.44 (0.09) 0.41–0.46	0.44 (0.00) 0.41–0.44	0.990 (0,985–0,994)	0	0	0.03 (−010–015),	0.963	0.981 (<0.001)	0.44 (0.17) (0.27–0.61)
Width	1.25 (0.08) 1.23–1.27	1.22 (0.07) 1.20–1.24	1.23 (0.02) 1.22–1.25	0.938 (0,892 to 0,963)	0.005	0.013	0,03 (−0.16–0.23)	0.019	0.893 (<0.001)	1.23 (0.17) (1.16–1.40)

*Abbreviations*: CI, confidence interval; ICC, Intraclass Correlation Coefficient; LoA, 95% limits of agreement; MDC, Minimum Detectable Change *r*, Pearson correlation coefficient; SD, standard deviation.

Analysis of reliability of the SL dimensions by caliper between inter-session first and second observer (Table 8) showed excellent inter-rater reliability (ICC_(1-1)_ = 0.825–0.998) with a strong correlation (*r* = 0.725–0.998; *P* < 0.001) for length, thickness and width measurements. In addition, there were not any inter-rater statistically significant differences (*P* > 0.05) between first and second observer for length and thickness, width measurements.

**Table 8:** Analysis of reliability of the Spring Ligament dimensions by caliper between inter-session first and second observer and normalized values.

Obserber	First Observer Intersession	Second Observer Intersession	Intersession							
Variables	Mean (SD) 95% CI	Mean (SD) 95% CI	Mean (SD) 95% CI	ICC_(1-1)_ (95% CI)	SEM	MDC	*LoA (95%CI)*	*P*-value	*r* (*P*-value)	Normalized values (95% CI)
Length	1.63 (0.40) 1.53–1.73	1.58 (0.32) 1.50–1.66	1.60 (0.03) 1.55–1.65	0.825 (0,711 to 0,894)	0.030	0.083	0.05 (−0.50–0.60)	0.444	0.725 (<0.001)	1.58 (0.62) (0.96–2.20)
Thicknesses	0.41 (0.10) 0.38–0.44	0.41 (0.10) 0.38–0.44	0.41 (0.10) 0.38–0.44	0,994 (0,991 to 0,996)	0.007	0.021	0.00 (−0.03–0.03)	0.999	0.989 (<0.001)	0.41 (0.19) (0.22–0.60)
Width	1.21 (0.10) 1.19–1.24	1.22 (0.11) 1.19–1.24	1.21 (0.00) 1.20–1.22	0.998 (0.997–0.999)	0	0	−0.003 (−0.02–0.01)	0.886	0.998 (<0.001)	1.21 (0.21) (1–1.42)

*Abbreviations*: CI, confidence interval; ICC, Intraclass Correlation Coefficient; LoA, 95% limits of agreement; MDC, Minimum Detectable Change; *r*, Pearson correlation coefficient; SD, standard deviation.

Analysis of reliability and correlation of the SL dimensions between inter-session US and caliper measurements for both observers (Table 9) showed an excellent inter-rater reliability (ICC_(1-1)_ = 0.911–0.966) with a strong correlation (*r* = 0.852–0.937; *P* < 0.001) for length, thickness and width measurements. In addition, there were not inter-session statistically significant differences (*P* > 0.05) between US and caliper measurements length, thickness and width measurements.

**Table 9:** Analyses of reliability and correlation of the SL dimensions between inter-session US and caliper measurements for both observers.

Variable	Intersesion Us Measurements Observer 1 and 2	Intersesion Caliper Measurements Observer 1 and 2	*ICC (*_*1-1*_ *) (95% CI)*	*LoA (95% CI)*	*r* (*P*-value)	*P*-value
	Mean (SD) 95% CI	Mean (SD) 95% CI				
Length	1.57 (0.32) 1.49–1.65	1.58 (0.32) 1.50–1.66	0.966 (0.943 to 0.979)	0.04 (−0.24–0.31)	0,937 (<0.001)	0.538
Thicknesses	0.44 (0.09) 0.41–0.46	0.41 (0.10) 0.38–0.44	0.911 0.8377 to 0.9495)	−0.03 (−0.15–010)	0,856 (<0.001)	0.151
Width	1.23 (0.02) 1.22–1.25	1.22 (0.11) 1.19–1.24	0.919 0.869 to 0.950)	−0.02 (−0.25–0.22)	0,852 (<0.001)	0.240

*Abbreviations*: CI, confidence interval; ICC, Intraclass Correlation Coefficient; LoA, 95% limits of agreement; MDC, Minimum Detectable Change; *r*, Pearson correlation coefficient; SD, standard deviation; SL, Spring ligament; US, ultrasound imaging.

**Figure 3:**
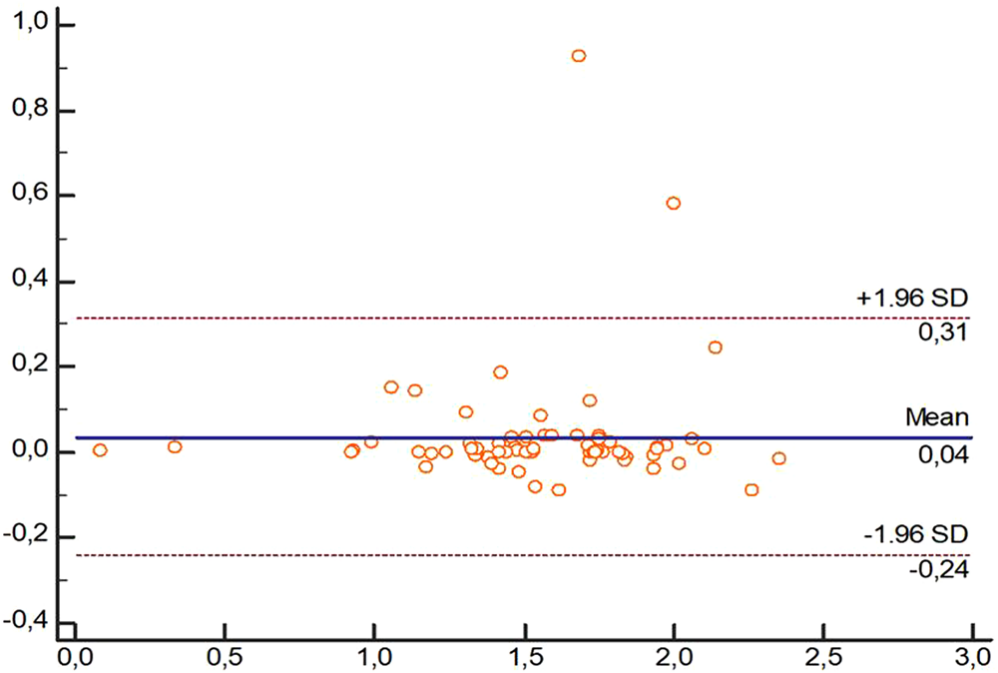
Bland-Altman plot comparing ultrasound and caliper devices to measure length of Spring Ligament in each foot specimen.

**Figure 4:**
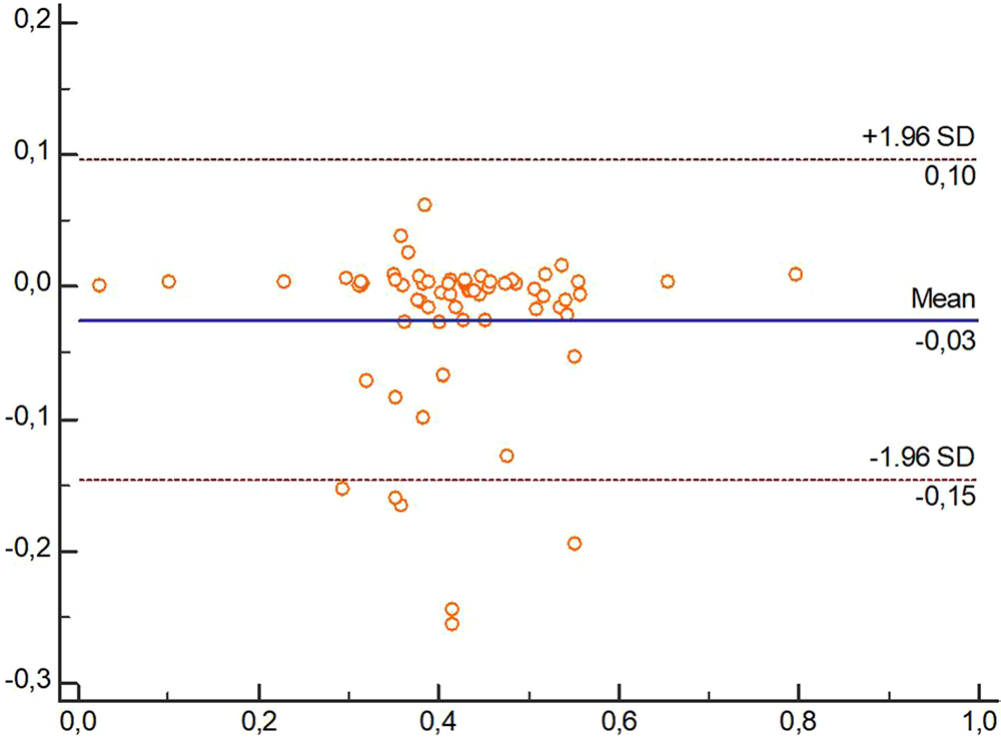
Bland-Altman plot comparing Ultrasound and caliper devices to measure thickness of Spring Ligament in each foot specimen.

The LoA (95% CI) of the measurements using both devices, US and caliper, showed values for all dimensions which tended to almost perfect agreement, showing no variability. Figures 2–4 showed the Brand-Altman plots for length, thickness and width dimensions, respectively, between US and caliper measurements. For each variable and almost every specimen, the difference between device´s means fell within the 95% CI of all measurements.

## Discussion

Several investigations about dimensions of the SL have used magnetic resonance imaging (MRI) to evaluate the anatomy of this structure in cadaveric feet^6,19,20^, specially Mengiardi *et al*. described accurately the SL complex in asymptomatic cadaveric feet.

**Figure 5:**
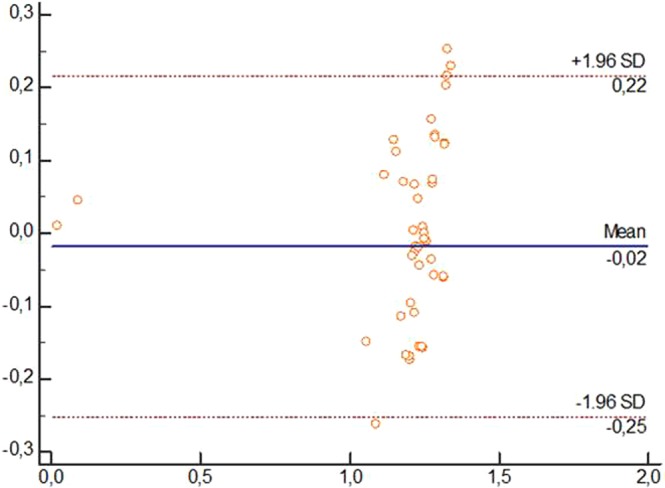
Bland-Altman plot comparing ultrasound and caliper devices to measure width of Spring Ligament in each foot specimen.

Despite both US and caliper measurements of SL dimensions have been previously carried out in cadavers^5–8^, our study may be considered as the first study showing an excellent inter-session and inter-rater reliability (ICC_US_ = 0.825–0.990; ICC_Caliper_ = 0.825–0.998; ICC_US vs Caliper_ = 0.911–0.966)^17^, absolute accuracy showing adequate SEM (SEM_US_ = 0–0.025 cm; SEM_Caliper_ = 0–0.030 cm), MDC (MDC_US_ = 0–0.069 cm; MDC_Caliper_ = 0–0.083 cm) and VN (VN_US_ = 0.44 [0.17] - 1.57 [0.62] cm; cm; VN_Caliper_ = 0.41 [0.19] - 1.58 [0.62] cm) values^12^, and almost perfect agreement according to the 95% CI LoA (LoA_US_ = −0.01 [−0.12–0.10] − 0,03 [−0.16–0.23]; LoA_Caliper_ = −0.003 [−0.02–0.01] − 0.05 [−0.50–0.60]; LoA_US vs Caliper_ = 0.03 [−0.15–010] − 0.04 [−0.24–0.31]) values and Bland-Altman plots distribution (Figs 3–5)^11,12^, as well as strong correlations (*r*_US_ = 0.893–0.989; *r*_Caliper_ = 0.725–0.998; ICC_US vs Caliper_ = 0.852–0.937)^18^ between caliper and US to determine all SL dimensions in cadaveric feet.

According to repeatability analyses^10–13^, our measurements showed good repeatability (*P*-value > 0.05) for the SL dimensions by US (Table 7), caliper (Table 8) and comparison between both tools (Table 9) between inter-session first and second observers values, except for SL width dimension measured with US (*P*-value = 0.019). Despite SL width dimensions should be considered with caution due to these US repeatability differences, to the authors’ knowledge, our study may be considered as the first research work providing reliability, absolute accuracy, correlation and repeatability for SL width dimension measured by US, due to prior US reliability studies mainly focused on SL length and thickness^5–8^.

In addition, MDC values for the SL dimensions, such as length (MDC_US_ = 0.069 cm versus MDC_Caliper_ = 0.083 cm), thickness (MDC_US_ = 0 cm versus MDC_Caliper_ = 0.021 cm) and width (MDC_US_ = 0.013 cm versus MDC_Caliper_ = 0 cm), showed that US measurements presented a higher absolute accuracy with lower MDC values than caliper measures for SL length and thickness dimensions, while caliper displayed greater absolute accuracy with lower MDC for SL width dimensions. According to MDC may be used as the change magnitude necessary to provide measuring confidence to be sure about these values are not the result secondary random variations or measurement errors^12^, these MDCs may be considered as cut-off reference values to determine SL dimensions modifications secondary to anatomic abnormalities^5–8^, ultrasound-guided invasive procedures^9^, and ligament injuries course after treatment^21,22^.

In accordance with our findings suggesting that these two techniques may be accurate for determining SL dimensions in human cadaveric feet, Harish *et al*. showed that US may be an effective imaging tool to evaluate SL abnormalities in patients with symptomatic posterior tibial tendon conditions compared to MRI as the gold standard tool^23^. In addition, Crim^24^ stated that MRI may be considered as the first-line evaluation procedure for the assessment of the SL conditions. Nevertheless, our study findings did not consider US and caliper measurements under SL conditions, while US and MRI have already been compared showing excellent findings^23^. As a future research line, we propose that both US and caliper reliability should be studied under SL pathologies.

The present study supported an ultrasound technical study for SL dimensions evaluations compared with caliper measures as gold standard which may be used as a reference for ultrasound-guided procedures in formaldehyde-embalmed human cadavers^9^. Future studies should consider these procedures in fresh-frozen cadavers as well as *in vivo* with healthy subjects and SL injured patients^21,22^.

Several limitations should be recognized regarding our approach for anatomical dissection and US procedures. Thus, we could not determinate the whole SL complex morphology and anatomic variations and further investigation is need in this field. First, only 2 observers were compared in the present study and future research studies should consider several observers for a better accuracy. Second, echogenicity changes could have modified the ability to perform the ultrasound measurements in ligament morphology, especially in the width dimensions showing a worse accuracy in the present study, given that the tissues have been infused with formalin for preservation due to this procedure can lead to asymmetric contraction of the tissue secondary to its anisotropic nature^9^.

## Conclusion

Both US and caliper could be recommended for all SL dimensions evaluation due to their excellent reliability and strong correlation in cadavers, although width dimensions should be considered with caution due to US repeatability differences.
